# GZ17-6.02 and palbociclib interact to kill ER+ breast cancer cells

**DOI:** 10.18632/oncotarget.28177

**Published:** 2022-01-11

**Authors:** Laurence Booth, Cameron West, Robert P. Moore, Daniel Von Hoff, Paul Dent

**Affiliations:** ^1^Department of Biochemistry and Molecular Biology, Virginia Commonwealth University, Richmond, VA 23298, USA; ^2^Genzada Pharmaceuticals, Sterling, KS 67579, USA; ^3^Physician-in-Chief, Distinguished Professor, Translational Genomics Research Institute (TGEN), Phoenix, AZ 85004, USA

**Keywords:** GZ17-6.02, palbociclib, 5FU, autophagy, breast cancer

## Abstract

GZ17-6.02 is presently undergoing clinical evaluation in solid tumors and lymphoma. The present studies were performed to define its biology in estrogen receptor positive breast cancer cells and to determine whether it interacted with palbociclib to enhance tumor cell killing. GZ17-6.02 interacted in an additive fashion with palbociclib to kill ER+ breast cancer cells. GZ17-6.02 and palbociclib cooperated to inactivate mTOR and AKT and to activate ULK1 and PERK. The drugs interacted to increase the expression of FAS-L and BAX, and to decrease the levels of MCL1, the estrogen receptor, and HDACs 1–3. Palbociclib activated ERBB3, an effect blocked by GZ17-6.02. GZ17-6.02 and palbociclib interacted to increase the expression of multiple toxic BH3 domain proteins and to reduce MCL1 and BCL-XL expression. Knock down of FAS-L reduced the lethality of [GZ17-6.02 + palbociclib]. GZ17-6.02 and palbociclib interacted to enhance autophagosome formation and autophagic flux. Knock down of Beclin1, ATG5, BAG3, eIF2α, toxic BH3 domain proteins or CD95 significantly reduced drug combination lethality. GZ17-6.02 and palbociclib increased the expression of Beclin1 and ATG5, effects blocked by knock down of eIF2α. The drugs also increased the phosphorylation of the AMPK and ATG13, effects blocked by knock down of ATM. Knock down of ATM or the AMPK, or expression of activated mTOR significantly reduced the abilities of GZ17-6.02 and palbociclib to enhance autophagosome formation and autophagic flux.

## INTRODUCTION

The therapeutic agent GZ17-6.02 has three components: curcumin, harmine and isovanillin [1–5]. It is currently undergoing phase I safety evaluation in cancer patients with solid tumors and lymphoma (NCT03775525). Our prior studies have shown that GZ17-6.02 kills a range of tumor cell types, including colorectal, pancreatic, hepatic, biliary, NSCLC, melanoma, sarcoma and actinic keratoses [1–5]. GZ17-6.02 interacted with 5FU *in vivo* to suppress the growth of colorectal tumor cells and *in vivo* to prolong animal survival [6]. The mechanisms by which GZ17-6.02 interacted with drugs such as 5FU, pemetrexed and dabrafenib/trametinib to kill tumor cells were similar: toxic autophagic flux, activation of death receptor signaling and mitochondrial dysfunction [1–6]. However, the biology of GZ17-6.02 in estrogen receptor (ER) positive breast cancer is presently unknown.

ER+ breast cancer is routinely treated with anti-estrogen drugs such as tamoxifen and aromatase inhibitors [7, 8]. More recently, the CDK4/6 inhibitor palbociclib is also used to treat ER+ advanced or metastatic breast cancer [9, 10]. Palbociclib can be combined with the pure anti-estrogen fulvestrant after disease progression on tamoxifen [11]. It also can be combined with aromatase inhibitors as a first line therapy [12]. Capecitabine is metabolized *in vivo* to become 5FU. Capecitabine as a monotherapy or in combination with other drugs is used to treat breast cancer that has loco-regional spread or is metastatic [13, 14]. The present studies were initiated to define the biology of GZ17-6.02 in ER+ breast cancer cells and to determine whether it interacted with the CDK4/6 inhibitor palbociclib to cause mammary tumor cell death, and if so, to define the mechanisms which are engaged/act to cause tumor cell killing. We then compared the biology of [GZ17-6.02 + palbociclib] to that of [GZ17-6.02 + 5FU] in ER+ breast cancer cells.

## RESULTS

**Figure 1:**
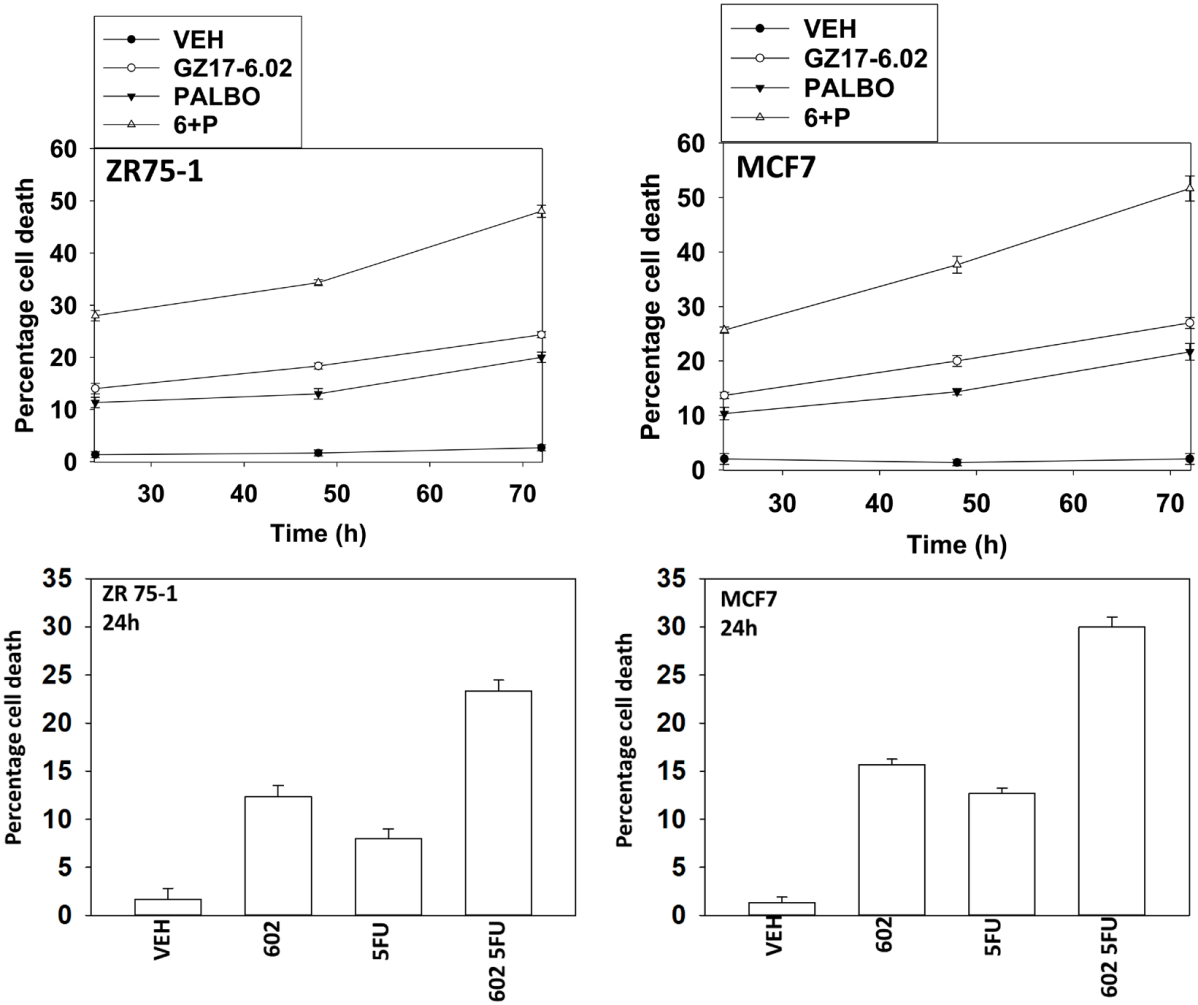
GZ17-6.02 interacts with 5FU and with palbociclib to kill ER+ breast cancer cells. ZR75-1 and MCF7 cells were treated with vehicle control, GZ17-6.02 (2 μM), 5FU (25 μM), palbociclib (100 nM) or the drugs in combination as indicated for 24 h, 48 h and 72 h. Cells were isolated, and viability at each time point determined by trypan blue exclusion. (*n* = 3 +/− SD).

Two established breast cancer therapeutic modalities are palbociclib and capecitabine (5FU). We performed studies to define the biology of GZ17-6.02 and palbociclib in ER+ breast cancer cells and then compared and contrasted those findings to the combination of GZ17-6.02 and 5FU, a drug combination we have tested in GI tumor cells [6]. We first determined whether these drugs interacted with GZ17-6.02 to kill estrogen receptor positive breast cancer cells (Figure 1; Supplementary Figure 1). GZ17-6.02 interacted in an arithmetically additive fashion with both 5FU and palbociclib to kill breast cancer cells. The amount of tumor cell killing increased over time, with the majority of cell killing observed over the first 48 h of incubation. As a single agent, GZ17-6.02 trended to be slightly more efficacious than the established drugs.

We then defined alterations in multiple signaling and protein expression parameters in cells treated with GZ17-6.02 and either palbociclib or 5FU. As single agents, GZ17-6.02 and/or palbociclib activated ATM and the AMPK and inactivated YAP, decreased expression of HDAC6 and increased phosphorylation of ATG13 S318 (Supplementary Figures 2 and 3). GZ17-6.02 and palbociclib cooperated to inactivate mTOR and AKT and to activate ULK1 and PERK. The drugs interacted to increase the expression of Beclin1, ATG5, FAS-L and variable multiple toxic BH3 domain proteins, and to decrease the levels of MCL1, the estrogen receptor, and HDACs 1-3. Palbociclib activated ERBB3, an effect blocked by GZ17-6.02. Inactivation of mTOR and AKT and activation of ULK1 with increased levels of Beclin1 and ATG5 predicts for greater autophagy. Increased levels of FAS-L, toxic BH3 domain proteins and MCL1 predicts for death receptor signaling and mitochondrial dysfunction. Reduced levels of the ER and HDAC6, and inactivation of YAP, predict slower growth and a reduced ability of cells to invade and metastasize.

As single agents, GZ17-6.02 and/or 5FU activated ATM and the AMPK and inactivated YAP/TAZ, decreased expression of HDAC6 and increased phosphorylation of ATG13 S318 (Supplementary Figure 4). This predicts for greater autophagy and less proliferation. GZ17-6.02 and 5FU also cooperated to inactivate mTOR and activate ULK1 and PERK. They interacted to increase the expression of Beclin1, ATG5, FAS-L and multiple toxic BH3 domain proteins and to reduce MCL1 and BCL-XL expression. The outcomes of these changes in cell biology and viability are very similar to those predicted for cells treated with GZ17-6.02 and palbociclib.

**Figure 2:**
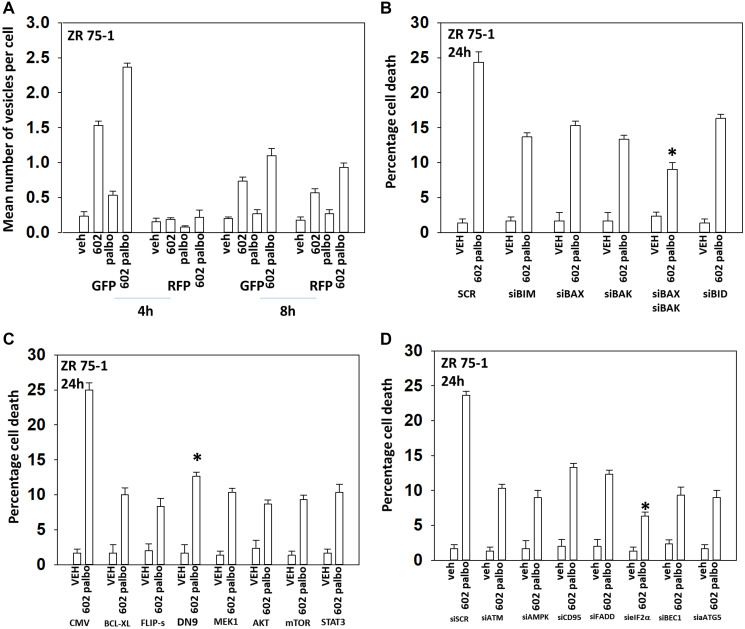
GZ17-6.02 and palbociclib interact to cause autophagosome formation and toxic autophagic flux in ZR75-1 cells. (**A**) ZR75-1 cells were transfected with a plasmid to express LC3-GFP-RFP. After 24 h, cells were treated with vehicle control, GZ17-6.02 (2 μM), palbociclib (100 nM) or the drugs combined for 4 h and for 8 h. The number of intense staining GFP+ and RFP+ punctae were determined randomly in at least 50 cells per time point and the mean number of punctae per cell determined (*n* = 3 +/− SD). (**B**) ZR75-1 cells were transfected with a scrambled siRNA or with validated siRNA molecules to knock down the expression of the indicated proteins. After 24 h, cells were treated with vehicle control, GZ17-6.02 (2 μM), palbociclib (100 nM) or the drugs combined for 24 h. Cells were isolated, and viability determined by trypan blue exclusion. (*n* = 3 +/− SD). ^*^ *p* < 0.05 less killing than all other treatments. (**C**) ZR75-1 cells were transfected with an empty vector plasmid or with plasmids to express BCL-XL, FLIP-s, dominant negative caspase 9, activated MEK1, activated AKT, activated mTOR or activated STAT3. After 24 h, cells were treated with vehicle control, GZ17-6.02 (2 μM), palbociclib (100 nM) or the drugs combined for 24 h. Cells were isolated, and viability determined by trypan blue exclusion. (*n* = 3 +/− SD). ^*^ *p* < 0.05 less killing than all other treatments. (**D**) ZR75-1 cells were transfected with a scrambled siRNA or with validated siRNA molecules to knock down the expression of the indicated proteins. After 24 h, cells were treated with vehicle control, GZ17-6.02 (2 μM), palbociclib (100 nM) or the drugs combined for 24 h. Cells were isolated, and viability determined by trypan blue exclusion. (*n* = 3 +/− SD). ^*^ *p* < 0.05 less killing than all other treatments.

**Figure 3:**
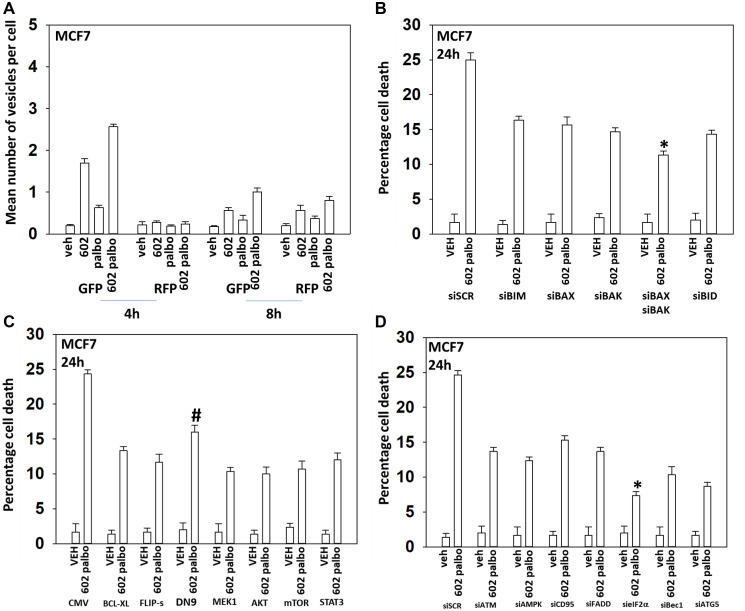
GZ17-6.02 and palbociclib interact to cause autophagosome formation and toxic autophagic flux in MCF7 cells. (**A**) MCF7 cells were transfected with a plasmid to express LC3-GFP-RFP. After 24 h, cells were treated with vehicle control, GZ17-6.02 (2 μM), palbociclib (100 nM) or the drugs combined for 4 h and for 8 h. The number of intense staining GFP+ and RFP+ punctae were determined randomly in at least 50 cells per time point and the mean number of punctae per cell determined (*n* = 3 +/− SD). (**B**) MCF7 cells were transfected with a scrambled siRNA or with validated siRNA molecules to knock down the expression of the indicated proteins. After 24 h, cells were treated with vehicle control, GZ17-6.02 (2 μM), palbociclib (100 nM) or the drugs combined for 24 h. Cells were isolated, and viability determined by trypan blue exclusion. (*n* = 3 +/− SD). ^*^ *p* < 0.05 less killing than all other treatments. (**C**) MCF7 cells were transfected with an empty vector plasmid or with plasmids to express BCL-XL, FLIP-s, dominant negative caspase 9, activated MEK1, activated AKT, activated mTOR or activated STAT3. After 24 h, cells were treated with vehicle control, GZ17-6.02 (2 μM), palbociclib (100 nM) or the drugs combined for 24 h. Cells were isolated, and viability determined by trypan blue exclusion. (*n* = 3 +/− SD). ^*^ *p* < 0.05 less killing than all other treatments. (**D**) MCF7 cells were transfected with a scrambled siRNA or with validated siRNA molecules to knock down the expression of the indicated proteins. After 24 h, cells were treated with vehicle control, GZ17-6.02 (2 μM), palbociclib (100 nM) or the drugs combined for 24 h. Cells were isolated, and viability determined by trypan blue exclusion. (*n* = 3 +/− SD). ^*^ *p* < 0.05 less killing than all other treatments.

In the following four Figures we next defined the mechanisms by which tumor cells died after GZ17-6.02 and palbociclib/5FU exposure. GZ17-6.02 and palbociclib interacted in an arithmetically additive fashion to increase autophagosome formation that was followed by autophagic flux (Figures 2 and 3). Cell killing was blocked by knock down of toxic BH3 domain proteins, particularly BAX combined with BAK, and by over-expression of BCL-XL. Knock down of Beclin1 or ATG5 and to a greater extent eIF2α was also protective. Knock down of CD95, FADD or BID, or over-expression of FLIP-s was also cyto-protective. Expression of dominant negative caspase 9 was less efficacious than over-expression of FLIP-s at preventing death. Collectively, this data argues that death receptor signaling, mitochondrial dysfunction and autophagy play interlocking roles in mediating cell death signals.

**Figure 4:**
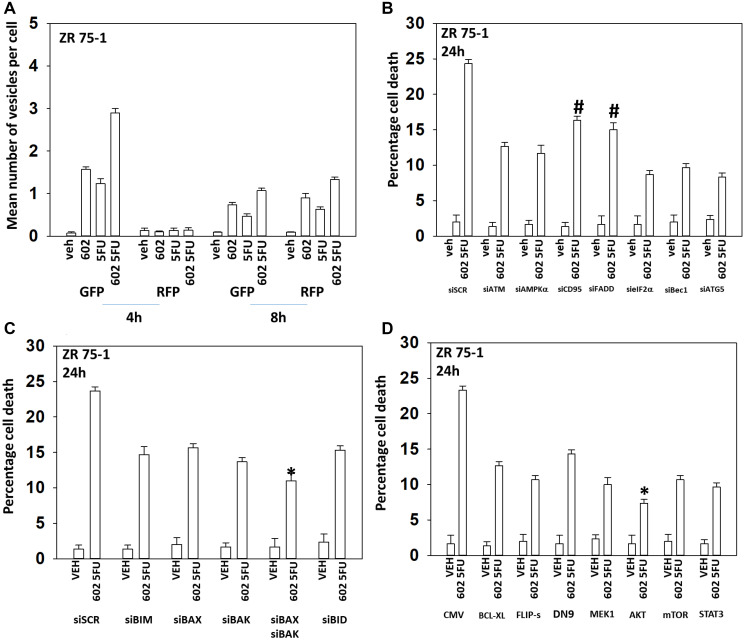
GZ17-6.02 and 5FU interact to cause autophagosome formation and toxic autophagic flux in ZR75-1 cells. (**A**) ZR75-1 cells were transfected with a plasmid to express LC3-GFP-RFP. After 24 h, cells were treated with vehicle control, GZ17-6.02 (2 μM), 5FU (25 μM) or the drugs combined for 4 h and for 8 h. The number of intense staining GFP+ and RFP+ punctae were determined randomly in at least 50 cells per time point and the mean number of punctae per cell determined (*n* = 3 +/− SD). (**B**) ZR75-1 cells were transfected with a scrambled siRNA or with validated siRNA molecules to knock down the expression of the indicated proteins. After 24 h, cells were treated with vehicle control, GZ17-6.02 (2 μM), 5FU (25 μM) or the drugs combined for 24 h. Cells were isolated, and viability determined by trypan blue exclusion. (*n* = 3 +/− SD). ^#^ *p* < 0.05 more killing than all other treatments except siSCR. (**C**) ZR75-1 cells were transfected with a scrambled siRNA or with validated siRNA molecules to knock down the expression of the indicated proteins. After 24 h, cells were treated with vehicle control, GZ17-6.02 (2 μM), 5FU (25 μM) or the drugs combined for 24 h. Cells were isolated, and viability determined by trypan blue exclusion. (*n* = 3 +/− SD). ^*^ *p* < 0.05 less killing than all other treatments. (**D**) ZR75-1 cells were transfected with an empty vector plasmid or with plasmids to express BCL-XL, FLIP-s, dominant negative caspase 9, activated MEK1, activated AKT, activated mTOR or activated STAT3. After 24 h, cells were treated with vehicle control, GZ17-6.02 (2 μM), 5FU (25 μM) or the drugs combined for 24 h. Cells were isolated, and viability determined by trypan blue exclusion. (*n* = 3 +/− SD). ^*^ *p* < 0.05 less killing than all other treatments.

**Figure 5:**
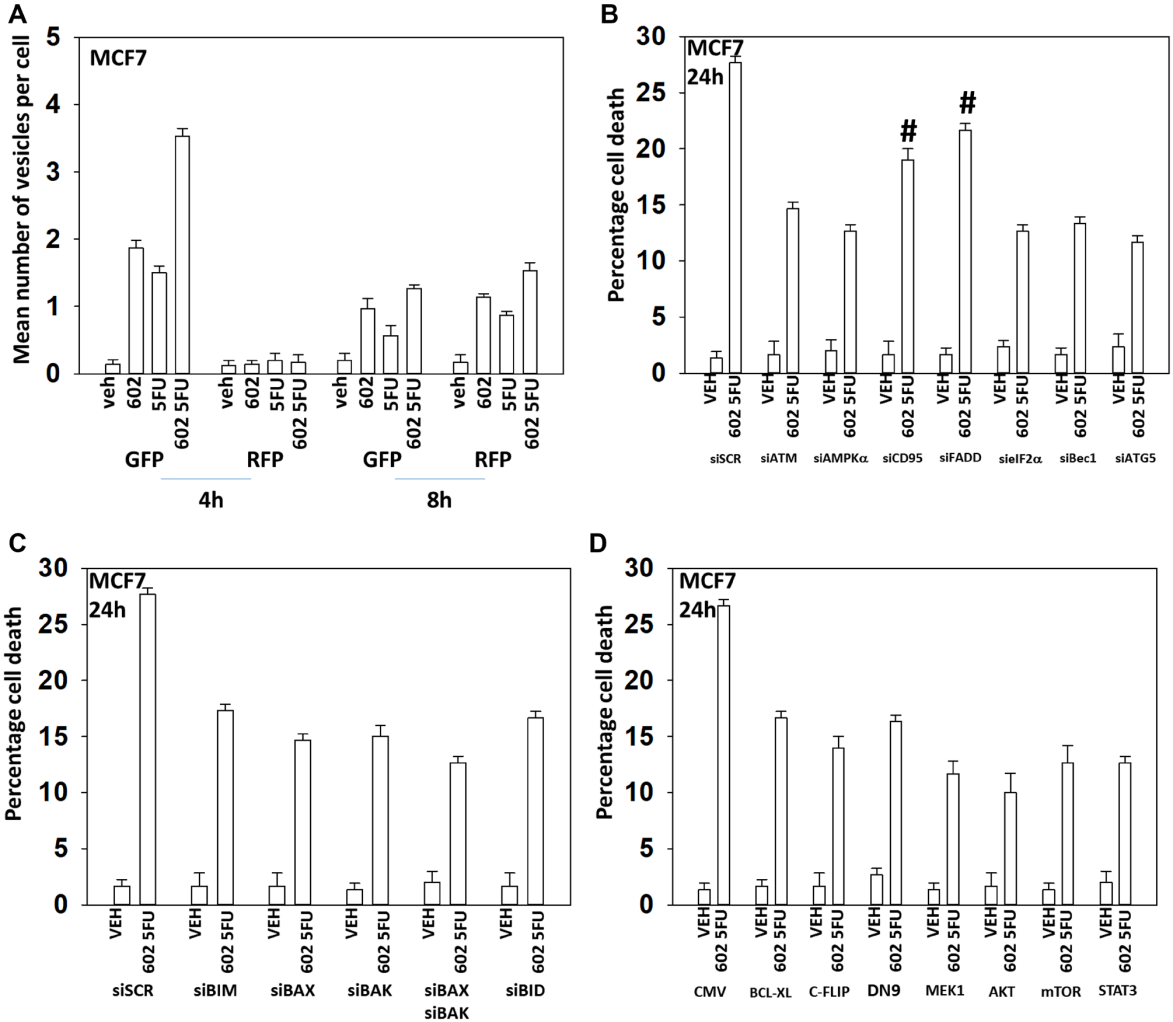
GZ17-6.02 and 5FU interact to cause autophagosome formation and toxic autophagic flux in MCF7 cells. (**A**) MCF7 cells were transfected with a plasmid to express LC3-GFP-RFP. After 24 h, cells were treated with vehicle control, GZ17-6.02 (2 μM), 5FU (25 μM) or the drugs combined for 4 h and for 8 h. The number of intense staining GFP+ and RFP+ punctae were determined randomly in at least 50 cells per time point and the mean number of punctae per cell determined (*n* = 3 +/− SD). (**B**) MCF7 cells were transfected with a scrambled siRNA or with validated siRNA molecules to knock down the expression of the indicated proteins. After 24 h, cells were treated with vehicle control, GZ17-6.02 (2 μM), 5FU (25 μM) or the drugs combined for 24 h. Cells were isolated, and viability determined by trypan blue exclusion. (*n* = 3 +/− SD). ^#^ *p* < 0.05 more killing than all other treatments except siSCR. (**C**) MCF7 cells were transfected with a scrambled siRNA or with validated siRNA molecules to knock down the expression of the indicated proteins. After 24 h, cells were treated with vehicle control, GZ17-6.02 (2 μM), 5FU (25 μM) or the drugs combined for 24 h. Cells were isolated, and viability determined by trypan blue exclusion. (*n* = 3 +/− SD). ^*^ *p* < 0.05 less killing than all other treatments. (**D**) MCF7 cells were transfected with an empty vector plasmid or with plasmids to express BCL-XL, FLIP-s, dominant negative caspase 9, activated MEK1, activated AKT, activated mTOR or activated STAT3. After 24 h, cells were treated with vehicle control, GZ17-6.02 (2 μM), 5FU (25 μM) or the drugs combined for 24 h. Cells were isolated, and viability determined by trypan blue exclusion. (*n* = 3 +/− SD). ^*^ *p* < 0.05 less killing than all other treatments.

GZ17-6.02 and 5FU also interacted in an arithmetically additive fashion to increase autophagosome formation that was followed by autophagic flux (Figures 4 and 5). Although knock down of CD95 or FADD was protective, it was less efficacious than knock down of Beclin1, ATG5, eIF2α or [BAX + BAK]. With this system, expression of activated AKT exhibited trended to be more protective than expression of activated MEK1/mTOR/STAT3. Thus, killing by the GZ17-6.02 5FU combination has overlapping but also separate mechanisms to that of GZ17-6.02 palbociclib.

**Figure 6:**
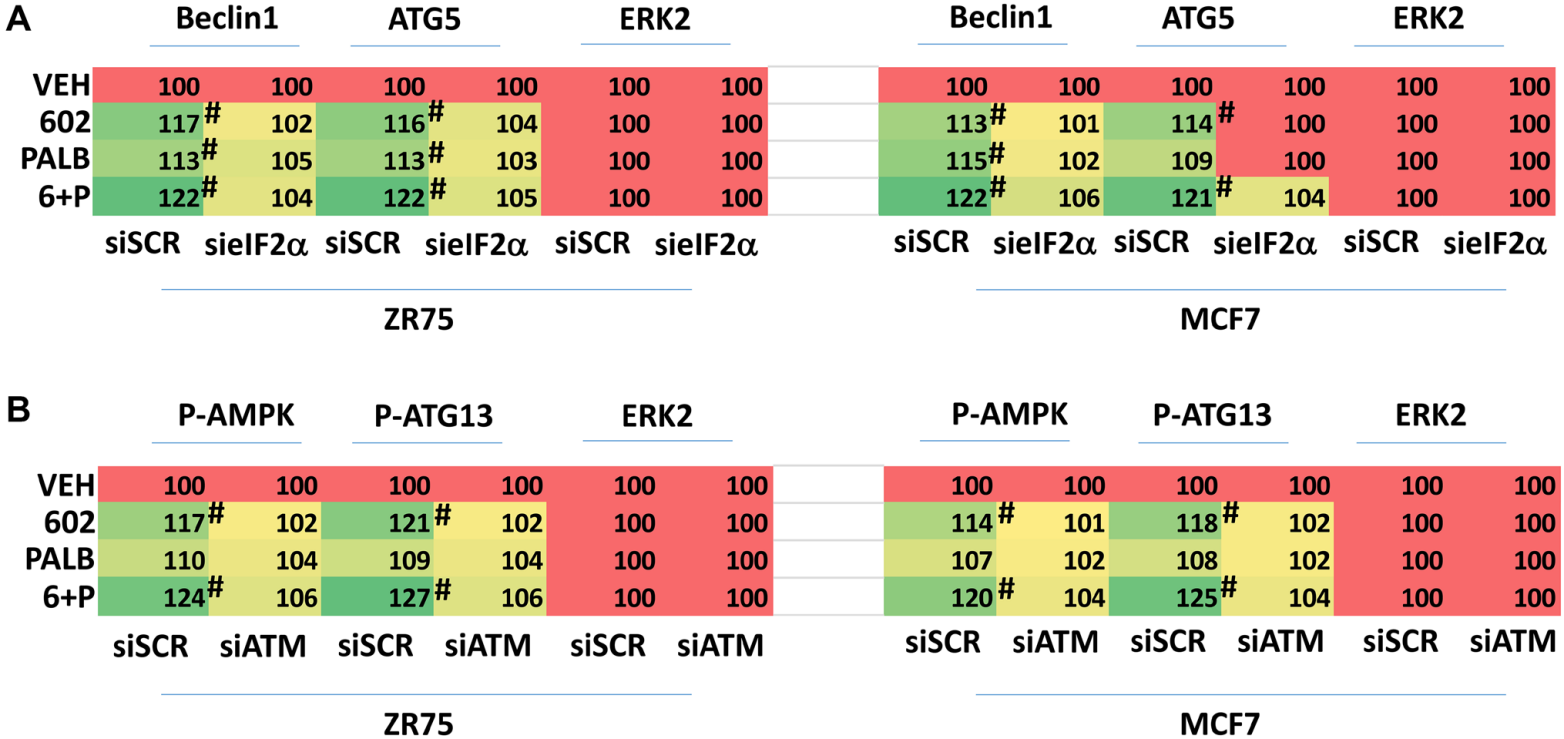
Increased expression of Beclin1 and ATG5 requires eIF2α and enhanced phosphorylation of the AMPK and ATG13 requires ATM. (**A**) ZR75-1 and MCF7 cells were transfected with a scrambled siRNA or with a validated siRNA to knock down the expression of eIF2α. After 24 h, cells were treated with vehicle control, GZ17-6.02 (2 μM), palbociclib (100 nM) or the drugs in combination as indicated for 6 h. At each time point the cells were fixed in place and immunostaining performed to determine the expression and phosphorylation of the indicated proteins. (*n* = 3 +/− SD). ^#^ *p* < 0.05 greater than vehicle control. (**B**) ZR75-1 and MCF7 cells were transfected with a scrambled siRNA or with a validated siRNA to knock down the expression of ATM. After 24 h, cells were treated with vehicle control, GZ17-6.02 (2 μM), palbociclib (100 nM) or the drugs in combination as indicated for 6 h. At each time point the cells were fixed in place and immunostaining performed to determine the expression and phosphorylation of the indicated proteins. (*n* = 3 +/− SD). ^#^ *p* < 0.05 greater than vehicle control.

We next interlinked alterations in the expression and phosphorylation of key proteins to primary signals induced by GZ17-6.02. GZ17-6.02 and palbociclib increased eIF2α S51 phosphorylation and knock down of eIF2α prevented the drugs alone or in combination from increasing the expression of Beclin1 and ATG5 (Figure 6A). GZ17-6.02 and palbociclib increased the phosphorylation of ATM and knock down of ATM prevented the drugs alone or in combination from increasing the phosphorylation of AMPKα T172 and ATG13 S318 (Figure 6B). n.b., in Figures 2 and 3, knock down of either Beclin1 or ATG5, i.e., required for macro-autophagy, reduced the lethality of [GZ17-6.02 + palbociclib].

**Figure 7:**
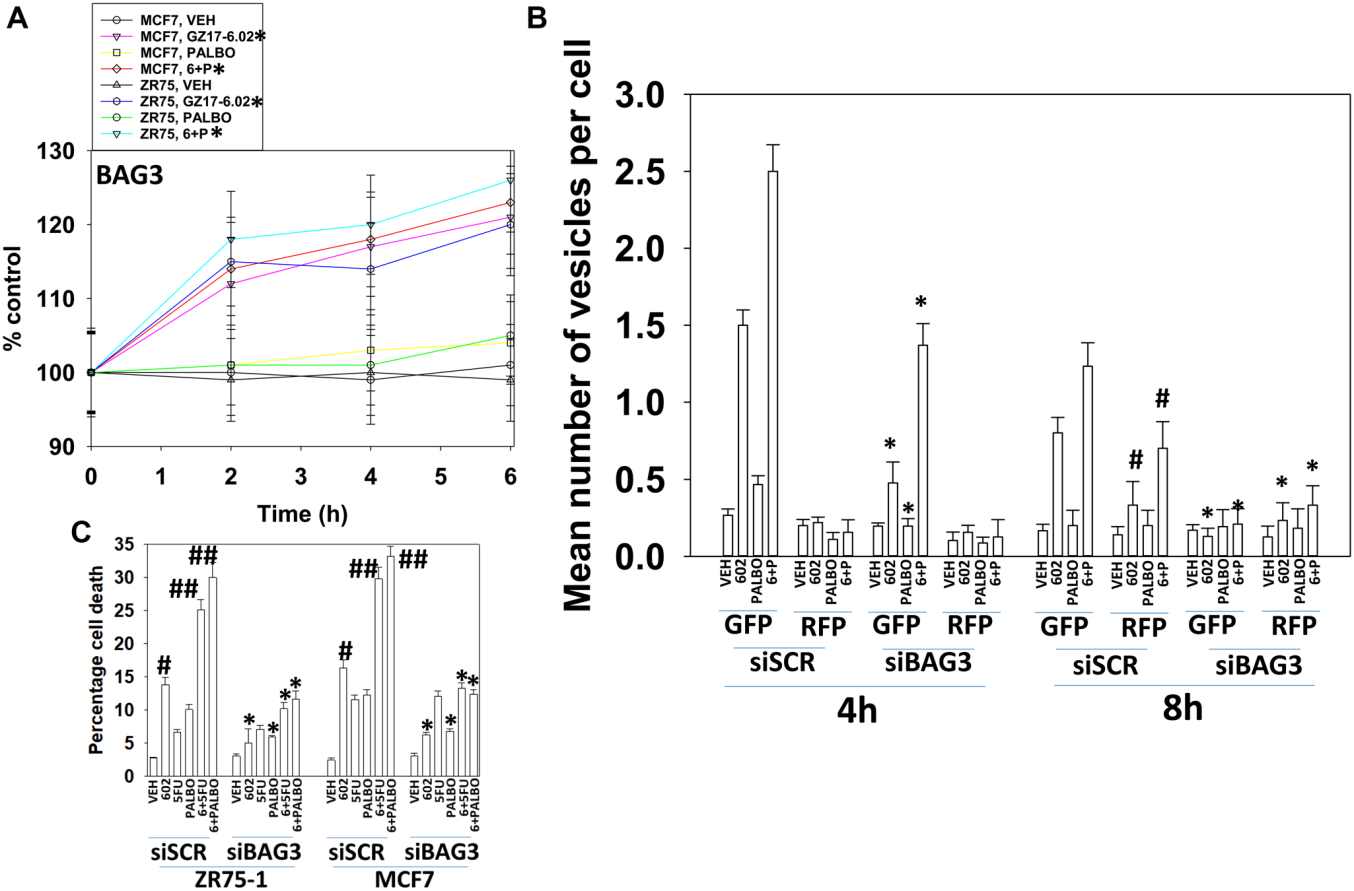
BAG3 is required for GZ17-6.02 -induced autophagy and tumor cell killing. (**A**) MCF7 and ZR75-1 cells were treated with vehicle control, GZ17-6.02 (2 μM), palbociclib (100 nM) or the drugs in combination as indicated for 2 h, 4 h and 6 h. At each time point the cells were fixed in place and immunostaining performed to determine the expression of BAG3 and ERK2. (*n* = 3 +/− SD). ^#^ *p* < 0.05 greater than vehicle control. (**B**) MCF7 cells were transfected with a scrambled siRNA or with an siRNA molecule to knock down BAG3, and in parallel transfected with a plasmid to express LC3-GFP-RFP. After 24 h, cells were treated with vehicle control, GZ17-6.02 (2 μM), palbociclib (100 nM) or the drugs combined for 4 h and for 8 h. The number of intense staining GFP+ and RFP+ punctae were determined randomly in at least 50 cells per time point and the mean number of punctae per cell determined (*n* = 3 +/−SD) ^*^ *p* < 0.05 less than corresponding value in siSCR cells; ^#^ *p* < 0.05 greater than corresponding value at 4 h. C. MCF7 and ZR75-1 cells were transfected with a scrambled siRNA or with an siRNA molecule to knock down BAG3. After 24 h, cells were treated with vehicle control, GZ17-6.02 (2 μM), palbociclib (100 nM) or the drugs combined for 24 h. Cells were isolated, and viability determined by trypan blue exclusion. (*n* = 3 +/− SD). ^*^ *p* < 0.05 less than corresponding value in siSCR cells; ^#^ *p* < 0.05 greater than vehicle control; ^##^ *p* < 0.05 greater than GZ17-6.02 alone value.

**Figure 8:**
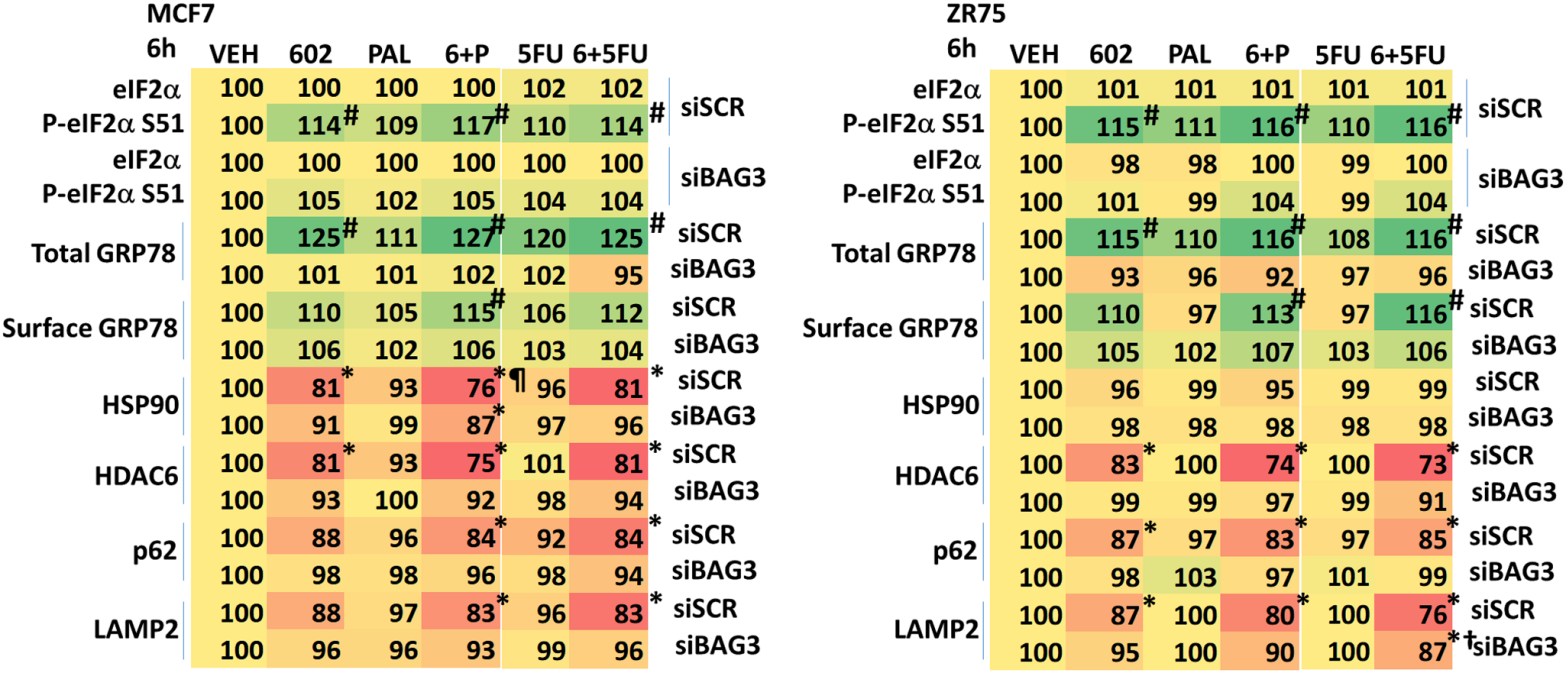
BAG3 is essential for the GZ17-6.02-induced eIF2α S51 phosphorylation and expression of GRP78. MCF7 and ZR75-1 cells were transfected with a scrambled siRNA or with an siRNA knock down BAG3. After 24 h, cells were treated with vehicle control, GZ17-6.02 (2 μM), palbociclib (100 nM), 5FU (25 μM) or the drugs in combination as indicated for 6 h. Cells were fixed in place and immunostaining performed to determine the expression and phosphorylation of the indicated proteins. (*n* = 3 +/−SD). ^#^ *p* < 0.05 greater than vehicle control; ^*^ *p* < 0.05 less than vehicle control; ^¶^ *p* < 0.05 less than corresponding value in siBAG3 cells; ^†^ *p* < 0.05 greater than corresponding value in siSCR cells.

The co-chaperone BAG3 plays an important role in assessing protein quality, as does GRP78. BAG3 over-expression has been linked in many studies to chemotherapy resistance, where an enhanced ability to form autophagosomes and initiate autophagic flux is protective against the anti-tumor actions of the chemotherapeutic drug [15, 16]. Treatment of cells with GZ17-6.02 increased BAG3 expression (Figure 7A). Knock down of BAG3 significantly reduced the ability of GZ17-6.02 to cause autophagosome formation and to kill breast cancer cells (Figure 7B, 7C). Knock down of BAG3 blocked drug-induced eIF2α S51 phosphorylation, the increase in GRP78 expression and the reduction of HSP90, HDAC6, p62 and LAMP2 levels (Figure 8). Thus, BAG3 is required for GZ17-6.02 -induced autophagy and tumor cell killing, and that BAG3 is required to generate a strong ER stress signal and reduction in HSP90 function.

**Figure 9:**
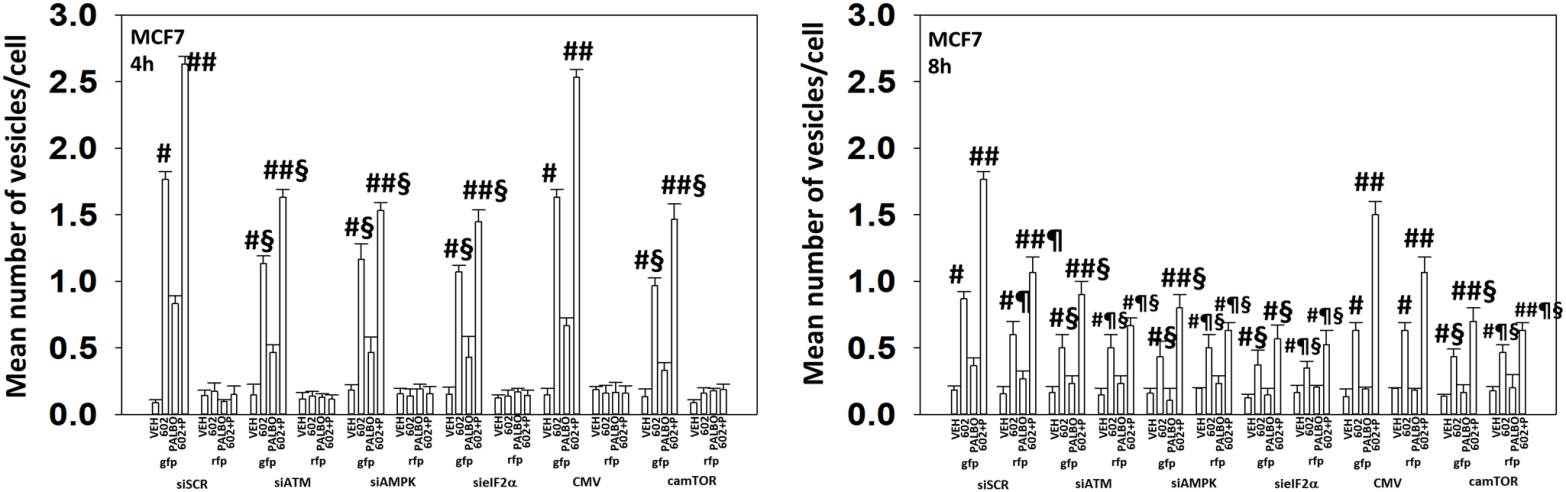
In MCF7 cells autophagic flux and the interaction between GZ17-6.02 and palbociclib requires expression of the AMPK and eIF2α. MCF7 cells were transfected with a plasmid to express LC3-GFP-RFP. In parallel, they were either transfected with a scrambled siRNA or with siRNA molecules to knock down the expression of ATM/AMPKα/eIF2α or transfected with an empty vector plasmid or with a plasmid to express activated mTOR. After 24 h, cells were treated with vehicle control, GZ17-6.02 (2 μM), palbociclib (100 nM) or the drugs combined for 4 h and for 8 h. The number of intense staining GFP+ and RFP+ punctae were determined randomly in at least 50 cells per time point and the mean number of punctae per cell determined (*n* = 3 +/−SD). ^#^ *p* < 0.05 greater than vehicle control; ^##^ *p* < 0.05 greater that GZ17-6.02 value as a single agent; ^§^ *p* < 0.05 less than corresponding value in siSCR cells; ^¶^ *p* < 0.05 greater than corresponding value after 4 h of exposure.

**Figure 10:**
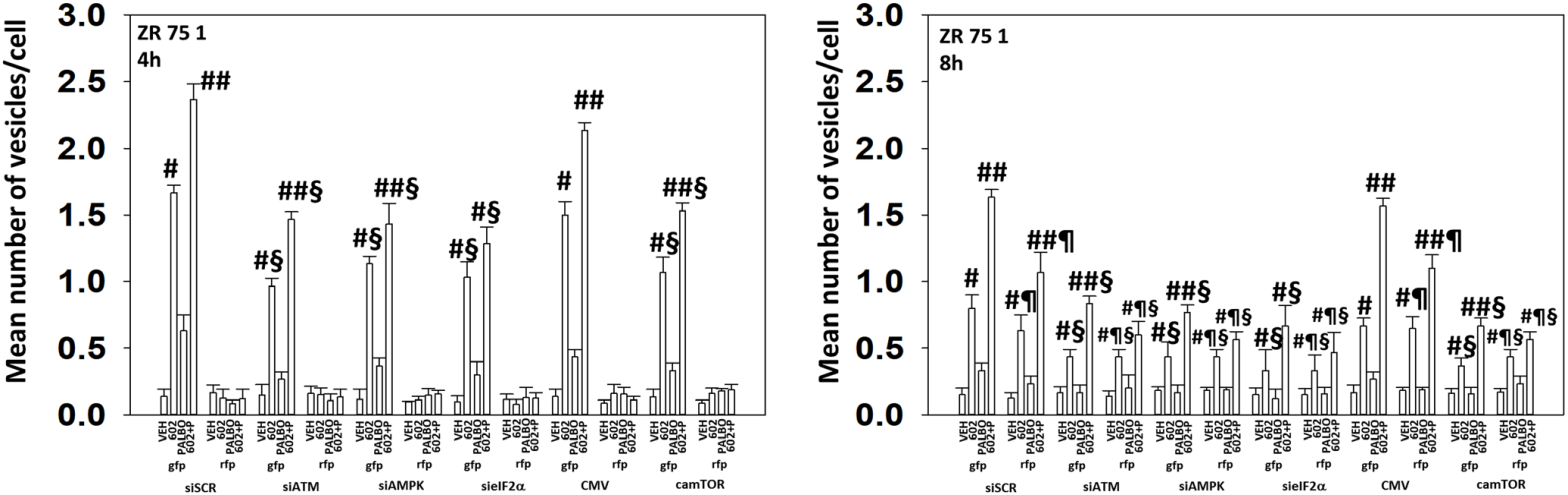
In ZR75-1 cells autophagic flux and the interaction between GZ17-6.02 and palbociclib requires expression of the AMPK and eIF2α. ZR75-1 cells were transfected with a plasmid to express LC3-GFP-RFP. In parallel, they were either transfected with a scrambled siRNA or with siRNA molecules to knock down the expression of ATM/AMPKα/eIF2α or transfected with an empty vector plasmid or with a plasmid to express activated mTOR. After 24 h, cells were treated with vehicle control, GZ17-6.02 (2 μM), palbociclib (100 nM) or the drugs combined for 4 h and for 8 h. The number of intense staining GFP+ and RFP+ punctae were determined randomly in at least 50 cells per time point and the mean number of punctae per cell determined (*n* = 3 +/−SD). ^#^ *p* < 0.05 greater than vehicle control; ^##^ *p* < 0.05 greater that GZ17-6.02 value as a single agent; ^§^ *p* < 0.05 less than corresponding value in siSCR cells; ^¶^ *p* < 0.05 greater than corresponding value after 4 h of exposure.

We next determined the importance of ATM, the AMPK, eIF2α and mTOR in autophagosome formation and autophagic flux. Knock down of either ATM, AMPKα or eIF2α significantly reduced the ability of drug-treated cells to form autophagosomes and to perform autophagic flux (Figures 9 and 10). As an internal control, we expressed activated mTOR which we have previously shown to reduce autophagy. Expression of activated mTOR reduced autophagosome formation and suppressed autophagic flux. Collectively, the data in Figures 9 and 10 strongly argues that activation of ATM and inactivation of eIF2α play key primary roles in promoting autophagosome formation caused by GZ17-6.02 and an ultimately toxic form of autophagy.

**Figure 11:**
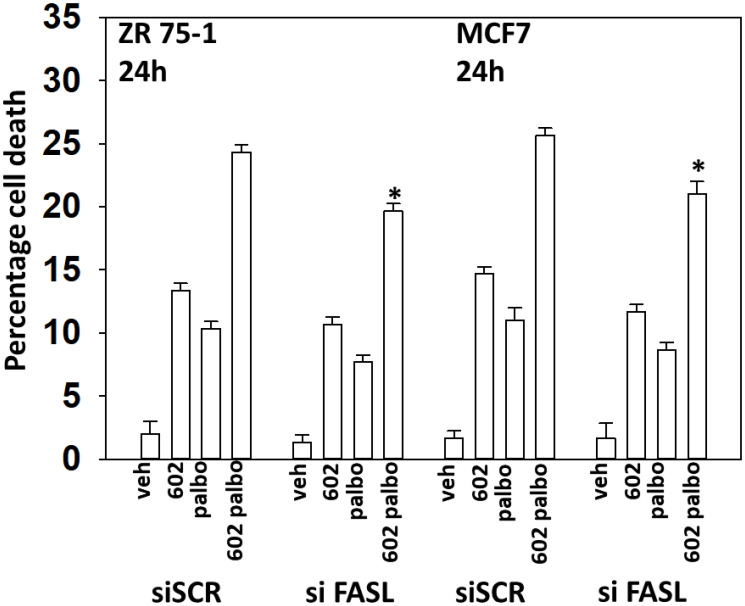
Drug combination-induced FAS-L expression plays a role in the toxic interaction between GZ17-6.02 and palbociclib. ZR75-1 and MCF7 cells were transfected with a scrambled siRNA or with an siRNA to knock down the expression of FAS-L. After 24 h, cells were treated with vehicle control, GZ17-6.02 (2 μM), palbociclib (100 nM) or the drugs combined for 24 h. Cells were isolated, and viability determined by trypan blue exclusion. (*n* = 3 +/−SD). ^*^ *p* < 0.05 less than corresponding value in siSCR cells.

One component of the cell killing process by the [GZ17-6.02 + palbociclib] combination was activation of CD95 death receptor signaling (the extrinsic apoptosis pathway). The drug combination, but not the individual drugs, increased the expression of FAS-L and we determined whether FAS-L production contributed to cell killing (Supplementary Figures 2 and 3). Knock down of FAS-L trended towards lowering cell death caused by GZ17-6.02 as a single agent but did significantly reduce cell killing by the drug combination (Figure 11). Thus GZ17-6.02 causes both ligand-independent and ligand-dependent CD95 signaling to cause tumor cell death.

## DISCUSSION

The present studies demonstrated that GZ17-6.02 interacted with the CDK4/6 inhibitor palbociclib to kill ER+ breast cancer cells. Our findings also confirmed that GZ17-6.02, as was observed in GI tumor cells, interacted with 5FU to kill breast cancer cells. The molecular mechanisms by which GZ17-6.02 interacted with either agent to cause tumor cell death were similar, and included death receptor signaling, autophagosome formation and autophagic flux, and mitochondrial dysfunction.

GZ17-6.02 and palbociclib increased the phosphorylation of AMPKα T172 and ATG13 S318 in an ATM-dependent fashion. The increase in Beclin1 and ATG5 expression after GZ17-6.02 and palbociclib exposure was prevented by knock down of eIF2α. Knock down of ATM, AMPKα or eIF2α significantly reduced the formation of autophagosomes and subsequent autophagic flux. Comparing our signaling data with the formation of autophagosomes and autolysosomes, it was apparent that significant levels of eIF2α S51 phosphorylation only occurred after autophagosome formation, i.e., the significant ER stress response was a secondary event to autophagy. Hence, the drug combination, downstream of ATM, promoted autophagosome formation which promoted eIF2α phosphorylation and the increased synthesis of Beclin1 and ATG5, proteins that will then act to facilitate greater levels of autophagosome formation.

The drugs interacted to increase the expression of FAS-L and BAX, and to decrease the levels of MCL1. Knock down of BAX significantly reduced cell killing as did over-expression of BCL-XL. Knock down of CD95 or FADD reduced cell killing and for the drug combination, so did knock down of FAS-L. Thus, death receptor signaling from CD95 is generated via ligand-dependent and ligand-independent mechanisms. As MCF7 cells do not express caspase 3, our data also suggests that CD95 signaling is causing non-apoptotic forms of tumor cell death.

Palbociclib activated ERBB3, an effect that was blocked by GZ17-6.02. We have recently observed that GI tumor cells treated with the clinically relevant drug combination of [neratinib + valproate] (NCT03919292) also cause surviving cells to evolve with higher ERBB3 signaling [17]. We have also shown that pancreatic tumors previously exposed to [sorafenib + vorinostat] (NCT02349867) evolved to activate the receptors ERBB1, ERBB2, ERBB3, c-MET and the intracellular kinase AKT [18]. Rapid compensatory activation of survival signaling or evolved long-term enhanced survival signaling are now well-recognized responses of tumor cells to targeted chemotherapies [19–21]. How GZ17-6.02 prevents palbociclib-induced ERBB3 activation is not yet understood. No activation of ERBB1/2/4 was observed, discounting ERBB3 trans-phosphorylation as the mechanism. SRC family enzymes can phosphorylate ERBB3 however, in our cells SRC activity trended to be reduced after drug treatment. Whether GZ17-6.02 causes activation of an ERBB3 protein tyrosine phosphatase will require studies beyond the scope of the present manuscript.

Capecitabine is a standard of care breast cancer therapeutic which is metabolized *in vivo* to 5FU. GZ17-6.02 and 5FU, as was observed for GZ17-6.02 and palbociclib, cooperated to inactivate mTOR and activate ULK1 and PERK. GZ17-6.02 and 5FU interacted to increase the expression of multiple toxic BH3 domain proteins and to reduce MCL1 and BCL-XL expression. Knock down of CD95, Beclin1, ATG5 or eIF2α significantly reduced tumor cell killing by the drug combination. Our data combining GZ17-6.02 and 5FU is congruent with our data in GI tumor cells, arguing for conserved mechanisms of drug action regardless of the tumor cell type being examined.

In both MCF7 and ZR75-1 cells, after 6 h of exposure, the GZ17-6.02/palbociclib drug combination reduced estrogen receptor expression. Loss of ER function will play a role reduced growth and in reduced viability [19]. ER+ mammary carcinoma cells have been shown to develop resistance to direct ER inhibitors through several mechanisms, most notably through activation of PI3K-AKT and MEK1/2-ERK1/2 signaling [22–29]. And one resistance mechanism upstream of the signaling pathways and growth factor receptors is increased expression of receptor ligands, such as heregulin [24, 25, 28]. In our studies we also observed the inactivation of STAT3 and STAT5, and enhanced STAT3 signaling has been linked to the development of estrogen resistance [26]. Collectively, our data demonstrate that GZ17-6.02 and palbociclib interact through multiple individual mechanisms to reduce cell viability. Future *in vivo* studies will be required to determine whether GZ17-6.02 and palbociclib interact to reduce tumor growth and prolong animal survival.

## MATERIALS AND METHODS

### Materials

The ZR75-1 and BT483 cell lines were obtained from the ATCC (Bethesda, MD). MCF7 cells derived from the original University of Michigan stock were kindly provided by Dr. K. Nephew (University of Indiana, Bloomington). Palbociclib and 5FU were purchased from Selleckchem (Houston, TX). All Materials were obtained as described in the references [1–5]. Trypsin-EDTA, DMEM, RPMI, penicillin-streptomycin were purchased from GIBCOBRL (GIBCOBRL Life Technologies, Grand Island, NY). Other reagents and performance of experimental procedures were as described [1–5]. Antibodies were purchased from Cell Signaling Technology (Danvers, MA); Abgent (San Diego, CA); Novus Biologicals (Centennial, CO); Abcam (Cambridge, UK); and Santa Cruz Biotechnology (Dallas, TX). Specific multiple independent siRNAs to knock down the expression of CD95, FADD, Beclin1, ATG5 AMPKa_1_, ATM, BIM, BAX, BAK, BID and eIF2α, and scramble control, were purchased from Qiagen (Hilden Germany). Control studies were presented showing on-target specificity of our siRNAs, primary antibodies, and our phospho-specific antibodies to detect both total protein levels and phosphorylated levels of proteins [1–6] (Supplementary Figure 5).

### Methods

All bench-side Methods used in this manuscript have been performed and described in the peer-reviewed references [1–5].

### Assessments of protein expression and protein phosphorylation

At various time-points after the initiation of drug exposure, cells are fixed in place using paraformaldehyde and using Triton X100 for permeabilization. Standard immunofluorescent blocking procedures are employed, followed by incubation of different wells with a variety of validated primary antibodies and subsequently validated fluorescent-tagged secondary antibodies are added to each well. The microscope determines the background fluorescence in the well and in parallel randomly determines the mean fluorescent intensity of 100 cells per well.

### Detection of cell death by trypan blue assay [1–5]

Cells were treated with vehicle control or with drugs alone or in combination for 24 h. At the indicated time points cells were harvested by trypsinization and centrifugation. Cell pellets were resuspended in PBS and mixed with trypan blue agent. Viability was determined microscopically using a hemocytometer. Five hundred cells from randomly chosen fields were counted and the number of dead cells was counted and expressed as a percentage of the total number of cells counted.

### Transfection of cells with siRNA or with plasmids [1–5]

Cells were plated and 24 h after plating, transfected. Plasmids to express FLIP-s, BCL-XL, dominant negative caspase 9, activated AKT, activated STAT3, activated mTOR and activated MEK1 EE were used throughout the study (Addgene, Waltham, MA). Empty vector plasmid (CMV) was used as a control. For siRNA transfection, 10 nM of the annealed siRNA or the negative control (a “scrambled” sequence with no significant homology to any known gene sequences from mouse, rat or human cell lines) were used.

### Assessments of autophagosome and autolysosome levels [1–5]

Cells were transfected with a plasmid to express LC3-GFP-RFP (Addgene, Watertown MA). Twenty-four hs after transfection, cells are treated with vehicle control or the drugs alone or in combination. Cells were imaged and recorded at 60X magnification 4 hs and 8 hs after drug exposure and the mean number of GFP+ and RFP+ punctae per cell determined from >50 randomly selected cells per condition.

### Data analysis

Comparison of the effects of various treatments was using one-way ANOVA for normalcy followed by a two tailed Student’s *t*-test with multiple comparisons. Differences with a *p*-value of < 0.05 were considered statistically significant. Experiments are the means of multiple individual data points per experiment from 3 independent experiments (± SD).

## SUPPLEMENTARY MATERIALS



## Abbreviations

ERK: extracellular regulated kinase
PI3K: phosphatidyl inositol 3 kinase
ca: constitutively active
dn: dominant negative
ER: endoplasmic reticulum
AMPK: AMP-dependent protein kinase
mTOR: mammalian target of rapamycin
JAK: Janus Kinase
STAT: Signal Transducers and Activators of Transcription
MAPK: mitogen activated protein kinase
PTEN: phosphatase and tensin homologue on chromosome ten
ROS: reactive oxygen species
CMV: empty vector plasmid
si: small interfering
SCR: scrambled
VEH: vehicle
602: GZ17-6.02
5FU: 5-flourouracil
PALB: palbociclib
PD-L1: programed death ligand 1
HDAC: histone deacetylase
