# Supplementary material for: GZ17-6.02 and palbociclib interact to kill ER+ breast cancer cells
Source: Oncotarget. 2022 Jan 11;13:92–104. doi: 10.18632/oncotarget.28177 (PMC8754587; doi:10.18632/oncotarget.28177)
Supplement: Supplementary file 1 [file oncotarget-13-28177-s001.pdf]

GZ17-6.02 and palbociclib interact to kill ER+ breast cancer cells

SUPPLEMENTARY MATERIALS

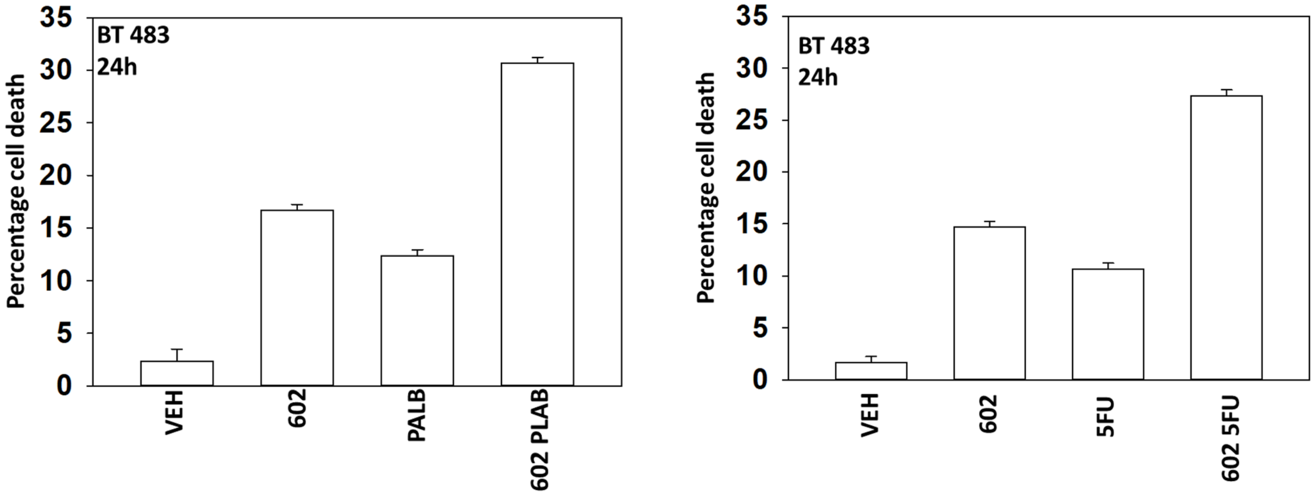

Supplementary Figure 1: GZ17-6.02 interacts with 5FU and with palbociclib to kill ER+ breast cancer cells. BT483 cells were treated with vehicle control, GZ17-6.02 (2 μM), 5FU (25 μM), palbociclib (100 nM) or the drugs in combination as indicated for 24 h. Cells were isolated, and viability determined by trypan blue exclusion. (n = 3 +/− SD).

ZR75, 3h	602	PALBO	6+P
ERBB1	99	99	97
P-ERBB1	101	101	99
ERBB2	99	99	99
P-ERBB2	92	96	89
ERBB3	100	100	100
P-ERBB3	100	115#	100
ERRB4	100	100	100
P-ERBB4	98	107	98
PDGFRβ	101	100	100
P-PDGFRβ	95	100	93
ATM	100	99	100
P-ATM	108	106	109
AMPKα	100	100	100
P-AMPKα	109	115#	122#
mTOR	100	101	101
P-S2448	88	97	87*
P-S2481	89	96	88
ULK1	101	100	100
P-757	93	96	87*
P-317	107	112	115#
eIF2α	100	99	99
P-eIF2α	110	110	112
PERK	100	101	100
P-PERK	111	111	114#
Beclin1	109	112	114#
ATG5	105	107	114#
ATG13	102	101	101
P-ATG13	103	113#	116#
GRP78	105	108	110
CHOP	106	107	112
PP1	99	99	99
NFκB	100	100	100
P-NFκB	101	100	98
c-SRC	102	101	99
Y416	100	101	100
Y527	105	99	107
c-MET	100	100	99
P-MET	100	101	105
CD95	100	100	100
FAS-L	103	108	110
JAK2	100	100	100
P-JAK2	97	100	97
c-KIT	100	100	100
P-KIT	94	100	94
STAT3	101	100	100
P-Y705	95	99	95
STAT5	100	100	100
P-Y694	95	100	95
AKT	101	100	100
P-T308	91	106	89
p70 S6K	100	100	100
P-p70 T389	97	100	97
ERK1/2	100	100	99
P-ERK	96	97	96
p38	100	100	99
P-p38	99	101	100
JNK1/2	99	100	103
P-JNK	102	90	95

ZR75, 3h	602	PALBO	6+P
LATS1/2	100	98	99
P-1079	103	101	106
P-909	107	98	108
YAP	98	98	99
P-109	108	101	109
P-127	114#	100	122#
P-397	106	98	111
TAZ	100	100	100
P-89	102	101	104
ERK2	100	100	100
HDAC1	90	101	90
HDAC2	97	99	97
HDAC3	88	96	85*
HDAC4	101	100	100
HDAC5	100	98	100
HDAC6	80*	99	75*
HDAC7	99	101	98
HDAC8	101	100	100
HDAC9	101	101	101
HDAC10	98	98	88
HDAC11	96	96	95
BCL-XL	92	94	88
MCL1	97	96	85*
BAX	101	106	107
BAK	106	107	108
BIM	106	109	111
NEDD4	99	100	98
PTEN	100	101	100
ER	94	100	93
PD-L1	90	100	89
PD-L2	101	100	100
MHCA	106	102	106
ODC	97	100	97
IDO1	97	96	96
Cyclin D	100	108	102
Cyclin E	99	103	100

ZR75, 6h	602	PALBO	6+P
ERBB1	100	100	100
P-ERBB1	98	100	97
ERBB2	100	100	101
P-ERBB2	94	98	92
ERBB3	100	99	100
P-ERBB3	100	116#	100
ERRB4	99	99	99
P-ERBB4	98	101	98
PDGFRβ	101	100	100
P-PDGFRβ	95	98	89
ATM	99	100	99
P-ATM	110	109	116#
AMPKα	99	100	100
P-AMPKα	112	117#	125#
mTOR	99	100	100
P-S2448	86*	98	79*
P-S2481	87*	98	86*
ULK1	99	100	100
P-757	89	97	85*
P-317	112	114#	117#
eIF2α	101	100	99
P-eIF2α	118#	109	125#
PERK	101	99	102
P-PERK	113#	111	116#
Beclin1	113#	118#	121#
ATG5	114#	114#	119#
ATG13	100	100	101
P-ATG13	107	121#	127#
GRP78	110	112	117#
CHOP	105	109	112
PP1	102	99	101
NFκB	101	101	100
P-NFκB	100	98	93
c-SRC	101	101	101
Y416	97	102	96
Y527	106	98	107
c-MET	101	101	100
P-MET	94	99	92
CD95	100	101	101
FAS-L	104	109	113#
JAK2	101	100	99
P-JAK2	97	99	97
c-KIT	100	100	100
P-KIT	95	100	95
STAT3	100	99	98
P-Y705	92	99	89
STAT5	100	99	91
P-Y694	93	99	91
AKT	100	101	100
P-T308	90	106	85*
p70 S6K	100	99	100
P-p70 T389	97	101	98
ERK1/2	100	100	100
P-ERK	98	99	98
p38	99	99	99
P-p38	99	100	101
JNK1/2	101	100	100
P-JNK	104	88	92

ZR75, 6h	602	PALBO	6+P
LATS1/2	101	99	100
P-1079	109	102	110
P-909	111	109	112
YAP	98	99	99
P-109	113#	105	114#
P-127	117#	106	119#
P-397	105	99	105
TAZ	101	102	101
P-89	102	101	103
ERK2	101	99	100
HDAC1	89	102	86*
HDAC2	93	95	91
HDAC3	87*	97	86*
HDAC4	100	100	100
HDAC5	100	101	101
HDAC6	77*	98	75*
HDAC7	99	102	99
HDAC8	101	99	100
HDAC9	100	101	101
HDAC10	100	100	93
HDAC11	98	100	98
BCL-XL	93	91	89
MCL1	95	91	84*
BAX	103	108	110
BAK	108	109	111
BIM	111	113#	115#
NEDD4	96	97	95
PTEN	101	101	102
ER	93	99	87*
PD-L1	87*	99	85*
PD-L2	101	104	101
MHCA	107	103	112
ODC	101	98	103
IDO1	100	100	102
Cyclin D	100	107	101
Cyclin E	98	105	97

Supplementary Figure 2: GZ17-6.02 and palbociclib cooperate to inactivate mTOR and AKT and to activate ULK1 and PERK in ZR75-1 cells. ZR75-1 cells were treated with vehicle control, GZ17-6.02 (2 μM), palbociclib (100 nM) or the drugs in combination as indicated for 3 h and for 6 h. At each time point the cells were fixed in place and immunostaining performed to determine the expression and phosphorylation of the indicated proteins. (n = 3 +/− SD). $^{*}p < 0.05$ less than vehicle control; $^{**}p < 0.05$ less than GZ17-6.02 alone value; $^{\#}p < 0.05$ greater than vehicle control; $^{\#\#}p < 0.05$ greater than GZ17-6.02 alone value.

MCF7, 3h	602	PALBO	6+P
ERBB1	99	99	98
P-ERBB1	90	100	88
ERBB2	99	100	99
P-ERBB2	100	99	99
ERBB3	102	101	101
P-ERBB3	86*	111	93¶
ERRB4	99	99	100
P-ERBB4	100	102	97
PDGFRβ	100	100	100
P-PDGFRβ	92	103	90¶
ATM	100	100	100
P-ATM	108	105	110
AMPKα	100	102	99
P-AMPKα	110	112	114#
mTOR	100	100	100
P-S2448	89	98	86*
P-S2481	91	98	88
ULK1	100	100	101
P-757	94	98	93
P-317	105	109	114#
eIF2α	100	99	99
P-eIF2α	109	107	112
PERK	100	101	100
P-PERK	106	109	111
Beclin1	110	113#	117#
ATG5	109	107	112
ATG13	101	100	101
P-ATG13	106	116#	120#
GRP78	112	114#	119#
CHOP	106	112	116#
PP1	107	98	108
NFκB	99	101	100
P-NFκB	97	100	89
c-SRC	100	100	101
Y416	96	100	96
Y527	113#	98	114#
c-MET	91	91	91
P-MET	92	100	93
CD95	100	101	101
FAS-L	102	108	113#
JAK2	98	99	97
P-JAK2	96	101	89
c-KIT	102	100	102
P-KIT	94	100	95
STAT3	99	100	100
P-Y705	98	98	93
STAT5	100	100	100
P-Y694	94	98	93
AKT	100	100	100
P-T308	92	96	82*
p70 S6K	99	99	99
P-p70 T389	97	105	100
ERK1/2	100	100	100
P-ERK	92	101	87*
p38	100	101	100
P-p38	98	97	99
JNK1/2	101	100	101
P-JNK	96	89	86*

MCF7, 3h	602	PALBO	6+P
LATS1/2	100	100	100
P-1079	110	109	110
P-909	107	99	102
YAP	100	99	100
P-109	102	106	108
P-127	109	102	111
P-397	107	96	102
TAZ	101	101	100
P-89	110	105	113#
ERK2	100	100	100
HDAC1	97	100	95
HDAC2	89	94	87*
HDAC3	94	97	87*
HDAC4	99	99	101
HDAC5	100	99	99
HDAC6	86*	100	82*
HDAC7	99	100	98
HDAC8	100	100	100
HDAC9	99	99	100
HDAC10	99	99	100
HDAC11	100	100	100
BCL-XL	96	99	96
MCL1	93	95	91
BAX	108	103	110
BAK	107	95	112
BIM	106	109	110
NEDD4	95	96	91
PTEN	101	103	104
ER	96	99	96
PD-L1	93	100	90
PD-L2	99	100	97
MHCA	110	102	114#
ODC	98	103	101
IDO1	98	97	97
Cyclin D	92	104	91¶
Cyclin E	99	104	100

MCF7, 6h	602	PALBO	6+P
ERBB1	100	101	101
P-ERBB1	79*	100	71*
ERBB2	99	100	100
P-ERBB2	99	100	101
ERBB3	100	100	100
P-ERBB3	90	114#	86#¶
ERRB4	101	100	100
P-ERBB4	98	106	98
PDGFRβ	99	99	100
P-PDGFRβ	84*	105	92¶
ATM	100	99	100
P-ATM	112	109	115#
AMPKα	100	100	100
P-AMPKα	112	117#	118#
mTOR	100	100	100
P-S2448	89	98	86*
P-S2481	89	96	88
ULK1	100	100	100
P-757	92	98	89
P-317	112	113#	116#
eIF2α	100	100	100
P-eIF2α	113#	110	115#
PERK	101	101	101
P-PERK	112	103	114#
Beclin1	112	117#	120#
ATG5	117#	114#	117#
ATG13	102	101	102
P-ATG13	108	118#	127#
GRP78	117#	115#	117#
CHOP	113#	116#	120#
PP1	109	99	109
NFκB	101	100	100
P-NFκB	88	99	87*
c-SRC	100	101	100
Y416	96	101	95
Y527	109	103	110
c-MET	99	99	98
P-MET	98	96	90
CD95	100	100	100
FAS-L	103	111	114#
JAK2	100	100	100
P-JAK2	95	100	88
c-KIT	101	102	100
P-KIT	93	100	91
STAT3	99	100	99
P-Y705	91	98	87*
STAT5	100	100	100
P-Y694	94	98	89
AKT	100	100	100
P-T308	89	107	84*
p70 S6K	100	100	100
P-p70 T389	85*	110	95¶
ERK1/2	100	100	100
P-ERK	89	99	77*
p38	102	103	100
P-p38	98	100	100
JNK1/2	101	100	100
P-JNK	99	90	84*

MCF7, 6h	602	PALBO	6+P
LATS1/2	99	100	99
P-1079	113#	112	114#
P-909	113#	99	105
YAP	99	99	99
P-109	113#	104	112
P-127	115#	104	115#
P-397	113#	99	114#
TAZ	102	100	101
P-89	111	103	114#
ERK2	100	100	100
HDAC1	86*	99	80*
HDAC2	88	95	86*
HDAC3	99	101	90
HDAC4	100	102	101
HDAC5	100	98	100
HDAC6	80*	101	76*
HDAC7	97	101	97
HDAC8	101	101	102
HDAC9	101	100	102
HDAC10	100	99	98
HDAC11	99	99	101
BCL-XL	90	94	87*
MCL1	87*	91	87*
BAX	107	95	113#
BAK	108	109	112
BIM	108	113#	118#
NEDD4	94	97	92
PTEN	103	103	106
ER	88	91	85*
PD-L1	93	98	90
PD-L2	98	98	98
MHCA	110	103	114#
ODC	97	101	96
IDO1	97	96	96
Cyclin D	98	108	101
Cyclin E	98	104	100

Supplementary Figure 3: GZ17-6.02 and palbociclib cooperate to inactivate mTOR and AKT and to activate ULK1 and PERK in MCF7 cells. MCF7 cells were treated with vehicle control, GZ17-6.02 (2 μM), palbociclib (100 nM) or the drugs in combination as indicated for 3 h and for 6 h. At each time point the cells were fixed in place and immunostaining performed to determine the expression and phosphorylation of the indicated proteins. (*n* = 3 +/− SD). $^{*}p < 0.05$ less than vehicle control; $^{**}p < 0.05$ less than GZ17-6.02 alone value; $^{\#}p < 0.05$ greater than vehicle control; $^{\#\#}p < 0.05$ greater than GZ17-6.02 alone value.

MCF7, 6h	602	5FU	6+5FU	MCF7, 6h	602	5FU	6+5FU
ERBB1	100	100	99	LATS1/2	99	98	97
P-ERBB1	80*	114#	72*	P-1079	118#	110	120#
ERBB2	-	-	-	P-909	109	103	112
P-ERBB2	-	-	-	YAP	99	101	99
ERBB3	100	99	100	P-109	117#	110	118#
P-ERBB3	86*	107	74*	P-127	113#	97	114#
ERRB4	101	101	100	P-397	106	102	108
P-ERBB4	99	107	96	TAZ	99	99	97
PDGFRβ	99	100	100	P-89	111	106	119#
P-PDGFRβ	87*	97	82*	ERK2	100	99	100
ATM	101	100	101	HDAC1	84*	99	79*
P-ATM	115#	121#	126#	HDAC2	87*	92	84*
AMPKα	100	101	101	HDAC3	89	101	86*
P-AMPKα	111	115#	118#	HDAC4	98	101	101
mTOR	101	101	101	HDAC5	99	103	104
P-S2448	93	91	87*	HDAC6	77*	102	72*
P-S2481	89	92	86*	HDAC7	97	98	97
ULK1	102	100	99	HDAC8	100	101	103
P-757	93	94	82*	HDAC9	101	104	102
P-317	113#	105	114#	HDAC10	100	100	98
eIF2α	100	100	100	HDAC11	103	105	106
P-eIF2α	113#	109	117#	BCL-XL	91	88	85*
PERK	99	96	97	MCL1	89	86*	83*
P-PERK	110	109	115#	BAX	105	108	113#
Beclin1	114#	118#	122#	BAK	106	110	112
ATG5	114#	114#	123#	BIM	104	108	110
ATG13	100	101	101	NEDD4	92	97	85*
P-ATG13	107	122#	126#	PTEN	102	103	104
GRP78	114#	119#	123#	ER	60*	76*	54*
CHOP	112	124#	127#	PD-L1	86*	100	74**
PP1	109	114#	114#	PD-L2	99	104	102
NFκB	101	99	112	MHCA	120#	120#	122#
P-NFκB	89	102	87*	ODC	102	104	102
c-SRC	101	102	102	IDO1	98	98	96
Y416	93	101	92				
Y527	105	97	106				
c-MET	102	111	112				
P-MET	92	100	87*				
CD95	102	102	102				
FAS-L	103	113#	117#				
JAK2	97	102	99				
P-JAK2	95	91	86*				
c-KIT	102	101	108				
P-KIT	93	93	92				
STAT3	100	100	100				
P-Y705	86*	91	82*				
STAT5	103	99	100				
P-Y694	95	96	89				
AKT	101	100	99				
P-T308	83*	79*	77*				
p70 S6K	99	100	99				
P-p70 T389	93	102	87*				
ERK1/2	100	100	100				
P-ERK	97	77*	72*				
p38	99	99	100				
P-p38	103	110	109				
JNK1/2	99	101	99				
P-JNK	103	104	106				

ZR-75, 6h	602	5FU	6+5FU	ZR-75, 6h	602	5FU	6+5FU
ERBB1	99	100	96	LATS1/2	101	99	100
P-ERBB1	97	100	97	P-1079	113#	105	113#
ERBB2	100	100	100	P-909	104	103	110
P-ERBB2	90	94	89	YAP	99	99	100
ERBB3	96	96	96	P-109	114#	99	115#
P-ERBB3	92	98	92	P-127	115#	108	118#
ERRB4	100	100	98	P-397	111	105	113#
P-ERBB4	99	105	96	TAZ	101	100	102
PDGFRβ	101	100	100	P-89	113#	105	120#
P-PDGFRβ	94	98	93	ERK2	100	100	100
ATM	101	101	102	HDAC1	89	101	90
P-ATM	112	117#	123#	HDAC2	94	98	92
AMPKα	100	101	101	HDAC3	88	97	85*
P-AMPKα	116#	116#	120#	HDAC4	99	101	100
mTOR	101	101	100	HDAC5	99	101	102
P-S2448	88	92	86*	HDAC6	80*	101	77*
P-S2481	90	93	86*	HDAC7	98	100	98
ULK1	100	101	99	HDAC8	102	103	104
P-757	94	96	87*	HDAC9	101	101	102
P-317	107	114#	117#	HDAC10	100	100	98
eIF2α	99	100	100	HDAC11	101	103	104
P-eIF2α	112	109	120#	BCL-XL	91	92	85*
PERK	99	96	98	MCL1	90	88	79*
P-PERK	120#	109	122#	BAX	109	113#	116#
Beclin1	113#	116#	118#	BAK	107	110	113#
ATG5	113#	112	116#	BIM	111	115#	121#
ATG13	100	101	101	NEDD4	93	95	82*
P-ATG13	107	119#	124#	PTEN	107	108	113#
GRP78	108	110	115#	ER	86*	95	85*
CHOP	105	108	111	PD-L1	92	101	89
PP1	100	99	101	PD-L2	101	100	100
NFκB	100	100	104	MHCA	110	109	112
P-NFκB	93	99	94	ODC	101	105	103
c-SRC	100	100	100	IDO1	103	102	104
Y416	97	101	97				
Y527	106	99	105				
c-MET	101	102	104				
P-MET	95	100	95				
CD95	101	101	101				
FAS-L	104	111	116#				
JAK2	100	100	100				
P-JAK2	98	94	85#				
c-KIT	100	100	101				
P-KIT	96	96	95				
STAT3	100	99	100				
P-Y705	96	99	94				
STAT5	100	101	101				
P-Y694	96	94	92				
AKT	100	100	99				
P-T308	92	92	91				
p70 S6K	102	101	102				
P-p70 T389	97	100	98				
ERK1/2	99	100	100				
P-ERK	98	82*	81*				
p38	99	100	100				
P-p38	100	105	107				
JNK1/2	100	100	100				
P-JNK	104	105	105				

BT483, 6h	602	5FU	6+5FU	BT483, 6h	602	5FU	6+5FU
ERBB1	99	100	100	LATS1/2	100	100	100
P-ERBB1	98	100	99	P-1079	113#	107	117#
ERBB2	98	100	100	P-909	103	103	109
P-ERBB2	100	95	92	YAP	99	99	100
ERBB3	99	100	100	P-109	116#	109	119#
P-ERBB3	94	98	91	P-127	119#	107	123#
ERRB4	101	100	100	P-397	117#	113#	121#
P-ERBB4	100	105	97	TAZ	100	100	101
PDGFRβ	100	101	99	P-89	115#	112#	117#
P-PDGFRβ	94	98	93	ERK2	100	100	100
ATM	101	100	102	HDAC1	97	102	93
P-ATM	110	117#	121#	HDAC2	94	100	94
AMPKα	102	100	100	HDAC3	97	100	94
P-AMPKα	111	108	117#	HDAC4	100	101	101
mTOR	101	100	100	HDAC5	100	100	101
P-S2448	94	98	88	HDAC6	87*	99	80*
P-S2481	92	92	89	HDAC7	99	100	100
ULK1	100	100	101	HDAC8	99	99	101
P-757	93	96	85*	HDAC9	99	101	100
P-317	107	114#	116#	HDAC10	101	100	100
eIF2α	100	100	100	HDAC11	99	98	98
P-eIF2α	108	102	113#	BCL-XL	87*	88	81*
PERK	99	98	99	MCL1	91	87*	83*
P-PERK	110	111	114#	BAX	106	109	112
Beclin1	114#	121#	122#	BAK	109	112	114#
ATG5	114#	112	117#	BIM	104	109	118#
ATG13	101	102	101	NEDD4	90	95	84*
P-ATG13	107	121#	126#	PTEN	106	105	108
GRP78	109	110	116#	ER	91	99	89
CHOP	105	108	111	PD-L1	92	101	90
PP1	108	99	109	PD-L2	101	103	100
NFκB	100	100	100	MHCA	109	108	117#
P-NFκB	94	101	92	ODC	100	105	102
c-SRC	101	102	101	IDO1	103	103	105
Y416	95	101	95				
Y527	106	98	107				
c-MET	100	101	101				
P-MET	94	100	94				
CD95	100	100	100				
FAS-L	104	108	116#				
JAK2	100	99	100				
P-JAK2	98	96	85*				
c-KIT	100	100	100				
P-KIT	96	96	93				
STAT3	100	99	100				
P-Y705	96	97	95				
STAT5	101	100	100				
P-Y694	94	94	92				
AKT	100	100	101				
P-T308	89	88	87*				
p70 S6K	102	101	98				
P-p70 T389	98	101	99				
ERK1/2	100	101	100				
P-ERK	97	84	82				
p38	100	98	99				
P-p38	102	106	111				
JNK1/2	101	98	99				
P-JNK	106	106	107				

Supplementary Figure 4: GZ17-6.02 and 5FU also cooperate to inactivate mTOR and activate ULK1 and PERK and increase the expression of multiple toxic BH3 domain proteins. ER+ breast cancer cells were treated with vehicle control, GZ17-6.02 (2 µM), 5FU (25 µM) or the drugs in combination as indicated for 6 h. At each time point the cells were fixed in place and immunostaining performed to determine the expression and phosphorylation of the indicated proteins. (n = 3 +/− SD). $^{*}p < 0.05$ less than vehicle control; $^{**}p < 0.05$ less than GZ17-6.02 alone value; $^{\#}p < 0.05$ greater than vehicle control; $^{\#\#}p < 0.05$ greater than GZ17-6.02 alone value.

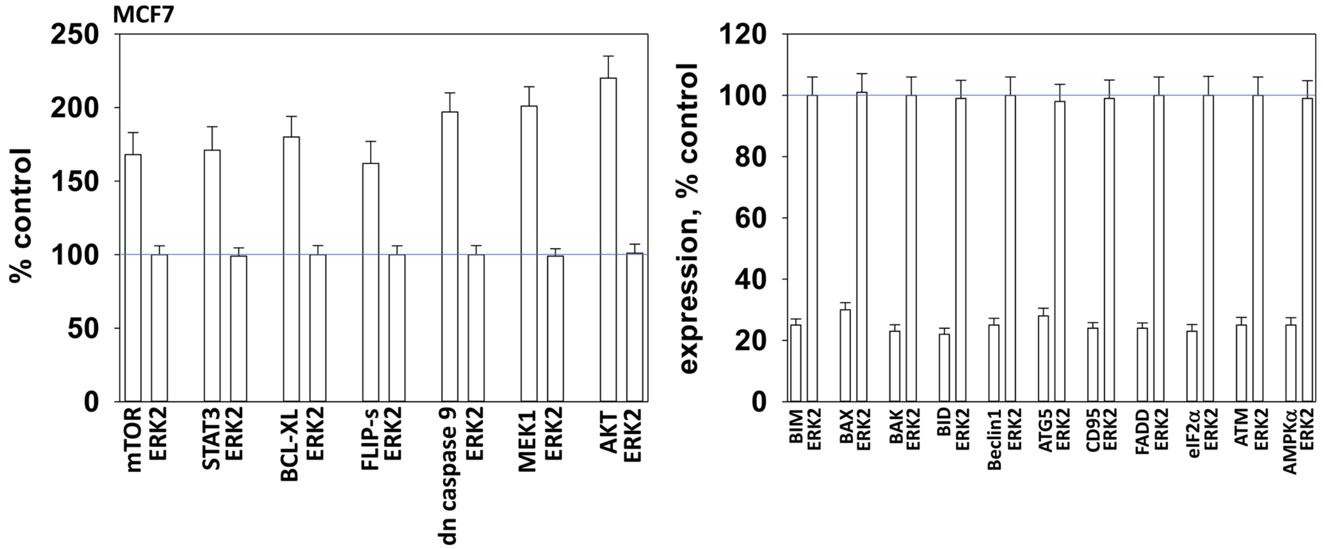

Supplementary Figure 5: Knock down and protein over-expression data in MCF7 cells. Cells were either transfected with a scrambled siRNA or an empty vector plasmid or transfected with plasmids to express the indicated proteins or with siRNA molecules to knock down the expression of the indicated proteins. 24 h after transfection cells were fixed in place and immunostaining performed to determine the expression level of each protein with ERK2 as an internal loading control (n = 3 +/− SD).
